# Supplementary material for: A Critical Analysis of the Drivers of Human Migration Patterns in the Presence of Climate Change: A New Conceptual Model
Source: Int J Environ Res Public Health. 2020 Aug 19;17(17):6036. doi: 10.3390/ijerph17176036 (PMC7504370; doi:10.3390/ijerph17176036)
Supplement: Supplementary file 1 [file ijerph-17-06036-s001.pdf]

Table S1: A descriptive table providing an overview of each variable included within the Malawi specific model. These variables were identified through an iterative process based on the Malawi context as well as data availability. Due to a lack of data on many variables some were omitted but may be considered in future work.

**Category**	**Variable**	**Brief description and justification for inclusion**
1. Climate change	Precipitation	Total precipitation, measured in mm per month, is a central aspect of weather and important indicator in long-term climatic changes.
	air temperature	Air temperature (degrees Celsius) is a key climate factors and as such forms a core part of any terrestrial climate change investigation.
	Soil moisture	Soil moisture (mm) represents the amount of plant extractable water (measured in mm each month) contained within the a grid of soil, measured in mm per volume (Wang-Erlandsson *et al.*, 2016; Abatzoglou *et al.*, 2018). As such, soil moisture presents an interesting metric to examine the impacts of climate change on crop yield and hence, upon people's livelihoods.
	Water surface runoff	It is hypothesized that high surface runoff events may be a primary cause of localized flooding which may lead either directly to displacement of persons, or to the damage of crop, property and life that may directly or indirectly impact upon a migration decision. Whilst this is not an indicator of climate change, it is highly related to precipitation measures and can be derived from general circulation models (GCMs) and as such is included within this category.
	Palmer Drought Severity Index (PDSI)	The Palmer Drought Severity Index is an index of relative dryness, initially created by Palmer in 1965 (Palmer, 1965) and as such provides an insightful measure in areas that are drought-vulnerable or are undergoing long-term drying, such as Malawi.<br>Droughts are hypothesized to be both a direct driver of movement (in extreme cases) as well as a key determinant of food security, due to the relationship between crop yield and drought (Government of Malawi, 2016).
1. Crop yield	As a subsistence farming society, it is hypothesized that in Malawi, climate change may impact food insecurity and people's livelihoods via impacting household crop yield.	

2. Food security	In any society, food security is a key component to a sustainable livelihood. Within the context of subsistence agrarian societies, such as much of rural Malawi, it is therefore hypothesized that food insecurity is a key determinant in the decision of whether to move and when. It is further hypothesized that, without food system interventions, climate change may increase food insecurity in the long term due to a gradual drying and warming of the climate which may have negative consequences for maize yields from traditional farming techniques that rely on rainfed irrigation (Niang *et al.*, 2014).	
4. Economic factors	Assets: fixed assets including land ownership, livestock and household goods	Household assets are an important determinant of household level food security because of the relationship between assets (as a proxy for wealth) and purchasing power (Hjelm, Mathiassen and Wadhwa, 2016). Furthermore, in climate migration literature, some studies find that household assets act as a confounder of migration, as saleable assets (such as livestock or furniture) presents an alternative adaptive approach to migration during lean times (Suckall, Fraser and Forster, 2017). Furthermore, some studies suggest that the more capital a household has in land, the less food insecure they are likely to be due to increased ability to grow food for both subsistence and as an income source (Peters, 2006; Skjeflo, 2013). Fixed assets (such as land, building materials, some livestock) may affect a household's willingness to move that requires partial or total abandonment of such investments and emotional attachment (Assa, Gebremariam and Mapemba, 2013). As such it is hypothesized within this thesis that household assets are relevant within the migration decision.
4. Economic factors	Income and access to credit	Income is an important determinant of household level food security because of the relationship between income and purchasing power. Richer households with more disposable cash are better able to purchase grain, farming equipment (such as fertiliser, seed and irrigation tools) or processed food (ready-to-eat) than poorer households. As such they are less vulnerable to food security during periods of poor environmental or economic conditions and therefore less vulnerable to forced migration.
4. Economic factors	Amount of land farmed	It is assumed that the more land available to an agent for subsistence farming, the higher the household crop yield and as such, the higher the household income, food security and resilience to future climate change.
4. Economic factors	Farming practices	Farming practices primarily refers to climate change adaptation schemes that may already be in place in certain parts of Malawi. For example, though much of

		Malawian agriculture is rain-fed, there are many localised irrigation schemes, both government and community planned to combat increases in local rainfall variability and increased periods of extreme rainfall conditions (Joshua *et al.*, 2016). In *dambo* (wetland) areas, adaptation strategies include increased management of water resources and uptake of government funded water pumps (Chidanti-Malunga, 2011; Joshua *et al.*, 2016). Other adaptive strategies include crop diversification, livelihood diversification (for example increased instances of fishing) (Chidanti-Malunga, 2011), circular migration (Suckall *et al.*, 2015), and perhaps even marital migration (Entwisle *et al.*, 2016; Myroniuk, 2017). Participation in subsidy schemes such as the FISP or SCTP may also impact a households economics and food security (Pauw and Thurlow, 2014; Government of Malawi, 2016).
4. Migration	Individual and household level movements.<br><br>District, regional and national (cross-border) movements	Measuring migration is highly challenging. It is important for studies to define the migration flow under scrutiny and select relevant spatial and temporal resolution that is most relevant to addressing the research question. These movements could be analysed at the individual, household (micro) levels, or district, regional and national level (macro).<br><br>In the Malawian context where movement is closely linked to food security, both district level and household level movements are of interest. Since food security typically varies seasonally with harvest and crop availability (Food and Agriculture Organization, 2015), seasonal movements are considered key, as are longer term movements in response to chronic or longer-term changes in food security. Short-term and circular movements are also thought to be relevant in climate change adaptation literature (Findley, 1994; Suckall *et al.*, 2015) though such small-scale movements are difficult to monitor due to data sparsity.
6. Mobility factors	Car / bike ownership	Car and bicycle ownership are a household asset that can be used as a proxy for identifying the wealth index of a household (Hjelm, Mathiassen and Wadhwa, 2016; National Statistics Office (NSO) [Malawi], 2016). Furthermore, such assets can be valuable in increasing an agent’s mobility and hence access to food from geographically further afield than less mobile households. Furthermore, such mobility increases the

		resilience of a household and as such may have downstream impacts upon a migration decision (Adger *et al.*, 2002). There are no studies that examine the relationship between vehicle or bicycle ownership with food security or propensity to migrate. However, it is intuitively hypothesized that better transportation methods make transportation easier and as such increase migration when other factors make the migration decision desirable.
6. Mobility factors	Proximity to main road	There are no current studies identified that study the relationship between road quality and quantity, with migration in Malawi. However there are studies that suggest that proximity to main roads impact upon other demographic aspects such as geospatial trends in HIV hot-spots in Malawi (Zulu, Kalipeni and Johannes, 2014), due to the effect of proximity to roads on facilitating travel. Furthermore, climate change, through physical extreme weather events, may contribute to road erosion and as such climate change may have an indirect impact upon migration based on the physical difficulty of moving due to poor roads (Arndt *et al.*, 2014).
6. Mobility factors	Proximity to city	Cities are often considered as pull factors attracting migration. There is evidence from gravity-model that suggest that proximity to city influences migration decisions (Henry, Boyle and Lambin, 2003; Mastrorillo *et al.*, 2016).
7. Sociodemographic factors	Age	Individual demographic characteristics of gender, age and marital status are well established demographic determinants of an individuals' vulnerability, and as such, to their propensity to migrate, and decisions regarding when and where, as well as the duration of migration.
7. Sociodemographic factors	Gender	
7. Sociodemographic factors	Marital status	
7. Sociodemographic factors	Employment type	Employment type is commonly associated with migration decisions and the type of migration undertaken based on human and financial capital that different types of employments provides. Employment status i.e. employed or unemployed (Mastrorillo *et al.*, 2016), industry for example arable, pastoral, ganyu or construction (Henry, Schoumaker and Beauchemin, 2004)and employment productivity markers that equates to income bracket such as size of farmland (Henry, Schoumaker and Beauchemin, 2004; Gray and Bilsborrow, 2013; Skjeflo, 2013) or level of

		ganyu income (Lewin, Fisher and Weber, 2012) are all found to be relevant variables in the migration decision.
7. Sociodemographic factors	Education level	Education is a well-documented confounder that is found to be relevant in migration decisions. Generally it is thought that the higher the level of education, the higher the likelihood of undertaking migration (Lewin, Fisher and Weber, 2012; Suckall, Fraser and Forster, 2017).
7. Sociodemographic factors	Number of dependents	Individual migration decisions often consider aspects such as age, gender, education and number of dependents as this affects mobility (ability to move) and alternative adaption options such as encouraging migration for work or marriage of some family members to provide remittances and reduce incumbency upon remaining members (Raleigh, 2011; Lewin, Fisher and Weber, 2012).
7. Sociodemographic factors	Social network	It is well established that social networks are an important dimension in a migration decision, with potential migrants more likely to move to areas where they have established connections (Myroniuk, 2017). In Malawi, social networks are intrinsic to migration decision for example through kinship, marriage, divorce, escape and widowhood (Myroniuk, 2017).<br>Social networks are also an important impact of migration, as migration can either strengthen or diminish social networks which can in turn have serious implications for wellbeing, access to healthcare and mental and physical health of migrants (Carr, 2005; McMichael, Barnett and McMichael, 2012).
